# Clinical outcome prediction of acute neurological patients admitted to the emergency department: Sequential Organ Failure Assessment score and modified SOFA score

**DOI:** 10.3389/fpubh.2023.1264159

**Published:** 2023-10-30

**Authors:** María I. Donoso-Calero, Ancor Sanz-García, Begoña Polonio-López, Clara Maestre Miquel, Carlos Durantez Fernández, Laura Mordillo-Mateos, Alicia Mohedano-Moriano, Rosa Conty-Serrano, Martin Otero-Agra, Cristina Jorge-Soto, José L. Martín-Conty, Francisco Martín-Rodríguez

**Affiliations:** ^1^Faculty of Health Sciences, Universidad de Castilla-la Mancha, Talavera de la Reina, Spain; ^2^Technological Innovation Applied to Health Research Group (ITAS), Faculty of Health Sciences, University of Castilla-La Mancha, Talavera de la Reina, Spain; ^3^Faculty of Nursing, Universidad de Valladolid, Valladolid, Spain; ^4^Faculty of Nursing, University of Castilla-La Mancha, Toledo, Spain; ^5^School of Nursing from Pontevedra, Universidade de Vigo, Pontevedra, Spain; ^6^Faculty of Nursing, University of Santiago de Compostela, Santiago de Compostela, Spain; ^7^Prehospital Early Warning Scoring-System Investigation Group, Valladolid, Spain; ^8^Faculty of Medicine, Universidad de Valladolid, Valladolid, Spain; ^9^Advanced Life Support, Emergency Medical Services (SACYL), Valladolid, Spain

**Keywords:** acute neurological disease, emergency department, mSOFA, SOFA, mortality

## Abstract

**Background:**

The aim of this study was to determine the ability of the Sequential Organ Failure Assessment score (SOFA) and modified SOFA score (mSOFA) as predictive tools for 2-day and 28-day mortality and ICU admission in patients with acute neurological pathology treated in hospital emergency departments (EDs).

**Methods:**

An observational, prospective cohort study in adults with acute neurological disease transferred by ambulance to an ED was conducted from 1 January 2019 to 31 August 2022 in five hospitals in Castilla-León (Spain). Score discrimination was assessed by the area under the curve (AUC) of the receiver operating characteristic (ROC) curve of the score.

**Results:**

A total of 640 adult patients with neurological disease were included. For the prediction of 2-day mortality (all-cause), mSOFA presented a higher AUC than SOFA (mSOFA = 0.925 vs. SOFA = 0.902). This was not the case for 28-day mortality, for which SOFA was higher than mSOFA (mSOFA = 0.852 vs. SOFA = 0.875). Finally, ICU admission showed that SOFA was higher than mSOFA (mSOFA = 0.834 vs. SOFA = 0.845).

**Conclusion:**

Both mSOFA and SOFA presented similar predictive ability, with mSOFA being the best predictor for short-term mortality and SOFA being the best predictor for medium-term mortality, as well as for ICU admission. These results in a cohort of patients with acute neurological pathology pave the way for the use of both predictive tools in the ED. The inclusion of these tools could improve the clinical assessment and further treatment of neurological patients, who commonly present the worst outcomes.

## 1. Introduction

Recent research has confirmed that the rates of patients attended at emergency departments (EDs) for acute neurological pathologies are approximately 20% of the total cases attended (1–3). Early intervention in the ED is crucial in the clinical evolution of patients with these conditions (4, 5). Its time-dependent component has been extensively studied, and protocols and hospital organization plans have been implemented to improve the interdisciplinary care of these cases (1, 6–8). Based on this, the use of risk scoring systems becomes necessary as a tool to harmonize the evaluation and standardize risk categories. These tools support the risk of early clinical deterioration assessment in patients with diverse conditions and in complex clinical settings. Due to their easy-to-use conception, they can be used in the prehospital setting, in the ED or in other hospital departments (9).

The research carried out with scores in recent years is extremely numerous, resulting in very heterogeneous scoring systems (10). For instance, the combined use with fast-processing biomarkers, especially lactate, improves their predictive ability (11, 12). The Sequential Organ Failure Assessment score (SOFA) is a wide-ranging scale with high implementation in intensive care units (ICUs) and EDs, which provides very adjusted information in various clinical situations (11). Because the original score included numerous laboratory determinations that hindered its use in many dynamic contexts, several modifications have been developed, for instance, quick-SOFA (qSOFA) or modified SOFA (mSOFA), enhancing its scope of application and streamlining the results (1, 5, 13). Particularly remarkable is the mSOFA score, which replaces the measurement of platelets and bilirubin with lactate (a biomarker that improves the predictive capacity of short- and medium-term mortality and adverse events) (14).

The literature in this field has demonstrated the adequate role of risk scoring systems as predictors of adverse events in various acute neurological pathologies (15–17). However, the role of SOFA and mSOFA in these patients has not been studied deeply. Thus, the primary objective was to validate this risk score as a predictive tool for 2- and 28-day mortality, and the second objective was to evaluate the risk score for ICU admission in patients with acute neurological pathology treated in the ED.

## 2. Methods

### 2.1. Study design and settings

An observational, prospective cohort study in adults with acute neurological disease transferred by ambulance to an ED was conducted from 1 January 2019 to 31 August 2022. Data collection took place in five hospitals in the Castilla-León region operated by the Public Health System of Castilla-León (SACYL): Segovia Hospital Complex (level II), Burgos University Hospital, Salamanca University Assistance Complex, Rio Hortega University Hospital, and Valladolid University Clinic (level III), complexity levels were assigned following national health system classification based in Hensher et al. (18). Ethical approval was obtained from the Research Ethics Committee of all participating centers (Ref. CEIC 2049, MBCA/dgc, PI 18–895, Ref. CEIm PI010-18, PI 2018 10–119). Registration of the study has been completed in the International Clinical Trials Registry Platform (ICTRP) of the World Health Organization.^1^ Informed consent was obtained from all patients or their legal guardians.

### 2.2. Participants

The study included adult patients (>18 years) who were collected uninterruptedly 24/7/365 and transferred by ambulance to the ED with an acute neurological disease diagnosis. Minors, pregnant females, individuals with acute psychiatric pathologies, those with terminal illness and specialist reports, cases of cardiorespiratory arrest upon ED arrival, patients without informed consent, or those lacking essential information for SOFA or mSOFA scores were excluded.

### 2.3. Outcomes

The main outcome was mortality at 2 and 28 days (all-cause and in-hospital). As a secondary outcome, ICU admission.

### 2.4. Measurement and data collection

The complete collected data included demographic variables (sex and age), initial evaluation (heart rate, respiratory rate, temperature, systolic, diastolic, and mean blood pressure, oxygen saturation, fraction of inspired oxygen and level of consciousness), and analytical variables (lactate, platelets, glucose, creatinine, and bilirubin). Additional information was recorded: hospital triage level (all the hospitals use the Manchester Triage system with levels from 1 to 5. Level 1: immediate response, level 2: very urgent, level 3: Urgent. The other two levels were not represented in our cohort and refer to low risk patients), pathology, hospital interventions (computerized axial tomography, ultrasound scan, surgery and coronary/neurovascular intervention) or hospital outcomes (hospitalization and ICU days).

The vital signs were obtained in a triage box by emergency registered nurses (ERNs): oxygen saturation, blood pressure, and heart rate; respiratory frequency was determined by monitoring ventilatory cycles through auscultation for 30 s (or 1 min in irregular breathing or extreme range cases); and neurological status was systematically monitored using the Glasgow Coma Scale (GCS). The analytical parameters (lactate, platelets, glucose, creatinine, and bilirubin) were obtained during the first 8 h of the patient’s stay in the ED in the first blood sample collected.

The mSOFA was calculated according to the score determined by Martín-Rodriguez et al. (14), where platelets and bilirubin were replaced by lactate, and the cutoffs for this metabolic biomarker were ≤ 2 mmol/L = 0 points, 2.1 to 3.0 mmol/L = 1 point, 3.1 to 4.0 mmol/L = 2 points, 4.1 to 6.0 mmol/L = 3 points, and > 6.0 mmol/L = 4 points. Table 1 shows a summary score resulting from the sum of points in each variable for SOFA and mSOFA.

**Table 1:** Sequential Organ Failure Assessment score (SOFA) and modified Sequential Organ Failure Assessment score (mSOFA).

		Points				
		0	1	2	3	4
Common items (SOFA and mSOFA)	Respiratory	PaO2/FiO2 > 400 SpO2/FiO2 > 302	PaO2/FiO2 < 400 SpO2/FiO2 < 302	PaO2/FiO2 < 300 SpO2/FiO2 < 221	PaO2/FiO2 < 200 SpO2/FiO2 < 142	PaO2/FiO2 < 100 SpO2/FiO2 < 67
	Renal, Creatinine (mg/dl)	<1.2	1.2–1.9	2.0–3.4	3.5–4.9	>5.0
	Neurologic, GCS (points)	15	13–14	10–12	6–9	<6
	Cardiovascular, MAP (mmHg)	≥70	<70	Dopamine≤5or Dobutamine (any dose)	Dopamine>5, Epinephrine≤0.1, or Norepinephrine≤0.1	Dopamine>15, Epinephrine>0.1, or Norepinephrine>0.1
SOFA items	Coagulation, Platelets (×10^3^/μL)	≥150	<150	<100	<50	<20
	Liver, Bilirubin (mg/dl)	<1.2	1.2–1.9	2.0–5.9	6.0–11.9	>12.0
mSOFA items	Metabolic, Lactate (mmol/L)	<2	2.1–3	3.1–4	4.1–6	>6

PaO2/FiO2 ratio, partial pressure of oxygen in arterial blood/fraction of inspired oxygen; SpO2/FiO2 ratio, pulse oximetry saturation/fraction of inspired oxygen ratio; GCS, Glasgow coma scale; MAP, mean arterial pressure.

### 2.5. Statistical methods

The collected data were stored in a database created using the software IBM SPSS Statistics for Windows version 20.0. (IBM Corp, Armonk USA). The database was reviewed for the detection of missing data, and no missing data were allowed, i.e., cases with missing data were excluded from the analysis (complete case study.). The final outcomes and predictors were completed by an independent investigator of each hospital through a review of the patients’ electronic medical records.

The univariate analysis used for cohort description and to report the association between predictors and the outcome was assessed by the Mann–Whitney U test or the chi-squared test, as appropriate. Categorical variables were described using absolute and relative frequencies. Quantitative variables were described as medians and interquartile ranges (IQR: 25^th^–75^th^ percentile) because they did not follow a normal distribution. Additionally, for quantitative variables, effect sizes (ES) were calculated with the Rosenthal *r* test and classified according to the following parameters: trivial (<0.2); small (0.2–0.5); moderate (0.5–0.8); large (0.8–1.3); and very large (≥1.3). For qualitative variables, ES was calculated with the Cramer V test and classified according to the following parameters: trivial (<0.1); small (0.1–0.3); medium (0.3–0.5); and large (≥0.5).

The score discrimination was assessed by the area under the curve (AUC) of the receiver operating characteristic (ROC) curve of the score in a validation cohort. The results from this analysis included the *p* value of the hypothesis test (H0: AUC = 0.5) and the 95% confidence interval (CI). Further statistical characteristics, such as the positive predictive value, negative predictive value, positive likelihood ratio, negative likelihood ratio, odds ratio, and diagnostic accuracy, were determined. Additionally, a calibration curve analysis was used to assess the reliability of the results.

All analyses were performed with XLSTAT BioMED software for Microsoft Excel version 14.4.0 (Microsoft Inc., Redmond, WA, USA) and IBM SPSS Statistics version 20.0 (IBM Corp., Armonk USA). In all tests, a confidence level of 95% and a *p* value below 0.05 were considered significant.

## 3. Results

**Figure 1:**
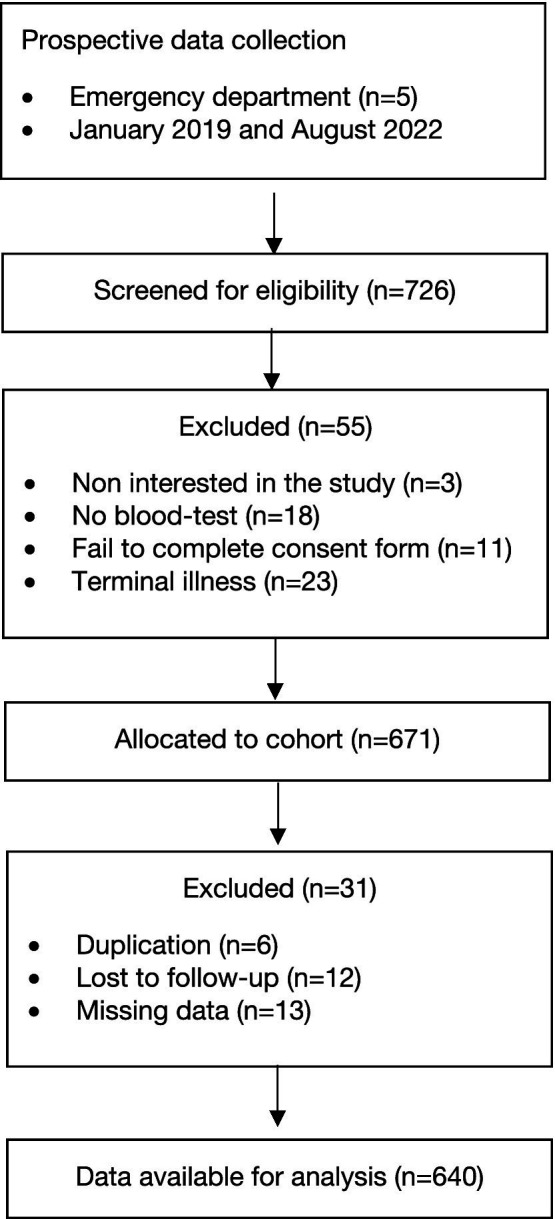
Flowchart.

Between 1 January 2019 and 31 August 2022, we recorded a total of 640 adult patients with neurological disease who were referred to the EDs of the five participating hospitals. Figure 1 shows the flowchart.

The median age was 67 years (IQR: 52–81 years), and 44% (281 patients) were females. The main reasons for medical check-up were seizures (186 cases, 29%), ischemic stroke (127 cases, 20%) and hemorrhage (118 cases, 18%), and their priority of care according to hospital triage was mainly level 2 (43%) or level 3 (41%). In total, 458 patients were hospitalized (72%), and ICU admission was required in 149 cases (23%). The mortality of the patients ranged from 9% (59 cases) within 2 days to 21% (132 cases) within 28 days (Table 2). The comparison of clinical variables between survivors and nonsurvivors showed that patients who died within 2 days presented higher mSOFA and SOFA scores. Supplementary Tables 1, 2 show the comparison of clinical variables for the other two outcomes.

**Table 2:** Comparison of patient variables recorded in the emergency department according to 2-day mortality.

Variables^1^	Total	Survivors	Nonsurvivors 2 days	*p* value and effect size^2^
Number	640 (100%)	581 (91%)	59 (9%)	
Demographic				
Age (years)	67 (52–81)	66 (51–80)	78 (65–83)	*p* < 0.001* (0.15) ^T^
Sex				
Male	359 (56%)	325 (56%)	34 (58%)	*p* = 0.80
Female	281 (44%)	256 (44%)	25 (42%)	
Initial evaluation				
Pulse (bpm)	83 (69–95)	82 (70–95)	86 (67–102)	*p* = 0.51
Respiratory rate (bpm)	15 (14–18)	15 (13–18)	15 (15–22)	*p* = 0.006* (0.11) ^T^
Temperature (°C)	36.0 (35.8–36.5)	36.0 (35.8–36.5)	36.0 (35.0–36.7)	*p* = 0.08
Systolic Blood Pressure (mmHg)	134 (117–155)	133 (117–152)	146 (105–173)	*p* = 0.22
Diastolic Blood Pressure (mmHg)	76 (65–86)	76 (65–86)	75 (60–97)	*p* = 0.91
Mean Blood Pressure (mmHg)	95 (84–108)	95 (84–107)	100 (73–122)	*p* = 0.48
SpO_2_ (%)	97 (95–99)	97 (95–99)	96 (91–100)	*p* = 0.11
SaFi	452 (284–467)	452 (354–467)	182 (96–243)	*p* < 0.001* (0.37) ^S^
Glasgow Coma Scale (total)	15 (10–15)	15 (12–15)	3 (3–7)	*p* < 0.001* (0.43) ^S^
Eye Opening Response	4 (3–4)	4 (3–4)	1 (1–1)	*p* < 0.001* (0.43) ^S^
Verbal Response	5 (3–5)	5 (4–5)	1 (1–1)	*p* < 0.001* (0.43) ^S^
Motor Response	6 (5–6)	6 (5–6)	1 (1–3)	*p* < 0.001* (0.45) ^S^
Lactate	2.3 (1.5–4.0)	2.1 (1.4–3.6)	4.9 (3.0–7.8)	*p* < 0.001* (0.26) ^S^
Platelets	213 (169–260)	215 (170–260)	199 (154–298)	*p* = 0.57
Glucose	132 (109–170)	131 (108–168)	150 (126–203)	*p* = 0.002* (0.13) ^T^
Creatinine	0.90 (0.74–1.15)	0.88 (0.74–1.12)	1.09 (0.75–1.76)	*p* = 0.002* (0.12) ^T^
Bilirubin	0.51 (0.45–0.66)	0.51 (0.45–0.64)	0.54 (0.46–1.06)	*p* = 0.004* (0.11) ^T^
Hospital triage				
Level 1 Immediate response	99 (16%)	69 (12%)	30 (51%)	*p* < 0.001* (0.31) ^M^
Level 2: Very urgent	276 (43%)	252 (43%)	24 (41%)	*p* = 0.69
Level 3: Urgent	265 (41%)	260 (45%)	5 (8%)	*p* < 0.001* (0.21) ^S^
Pathology				
Seizures	186 (29%)	185 (32%)	1 (2%)	*p* < 0.001* (0.19) ^S^
Ischemic stroke	127 (20%)	121 (21%)	6 (10%)	*p* = 0.051
Hemorrhage	118 (18%)	83 (14%)	35 (59%)	*p* < 0.001* (0.34) ^M^
Infection	53 (8%)	45 (8%)	8 (14%)	*p* = 0.12
Confusion syndrome	44 (7%)	43 (7%)	1 (2%)	*p* = 0.10
Degenerative disease	23 (4%)	23 (4%)	0 (0%)	*p* = 0.12
Headache	21 (3%)	21 (3%)	0 (0%)	*p* = 0.14
Coma	21 (3%)	15 (3%)	6 (10%)	*p* = 0.002* (0.12) ^S^
Vertigo	18 (3%)	18 (3%)	0 (0%)	*p* = 0.17
Tumor	17 (3%)	15 (3%)	2 (3%)	*p* = 0.71
Neuromediated syncope	12 (2%)	12 (2%)	0 (0%)	*p* = 0.27
Hospital interventions				
CT-scan	527 (82%)	474 (82%)	53 (90%)	*p* = 0.11
Ultrasound scan	150 (23%)	133 (23%)	17 (29%)	*p* = 0.31
Surgery	41 (6%)	35 (6%)	6 (10%)	*p* = 0.22
Coronary/neurovascular interv.	57 (9%)	54 (9%)	3 (5%)	*p* = 0.28
Hospital outcomes				
Inpatients	458 (72%)	400 (81%)	58 (98%)	*p* < 0.001* (0.19) ^S^
Hospitalization days (inpatients)	7 (3–14)	8 (5–15)	1 (1–2)	*p* < 0.001* (0.54) ^M^
Intensive care unit	149 (23%)	113 (19%)	36 (61%)	*p* < 0.001* (0.29) ^S^
Mortality				
Day 28	132 (21%)	73 (13%)	–	–
EWS analyzed				
mSOFA	2 (1–5)	2 (1–4)	9 (7–11)	*p* < 0.001* (0.43) ^S^
SOFA	1 (0–4)	1 (0–3)	7 (6–9)	*p* < 0.001* (0.41) ^S^

^1^Values expressed as a total number (fraction) and medians (1st quartile-3rd quartile) as appropriate. Bracketed numbers indicate 95% confidence interval. ^2^The *p* values were calculated with the Mann–Whitney U test and Chi square test. Effect Size were calculated with the Rosenthal r test [Trivial ^T^ (< 0.2); Small ^S^ (0.2–0.5); Moderate ^M^ (0.5–0.8) and Cramer V test [Trivial ^T^ (< 0.1); Small ^S^ (0.1–0.3); Medium ^M^ (0.3–0.5)]. SpO2, Oxygen saturation; SaFi, pulse oximetry saturation/fraction of inspired oxygen ratio; CT-scan, computerized axial tomography. SOFA, Sequential Organ Failure Assessment; mSOFA, modified Sequential Organ Failure Assessment. *p < 0.05.

**Figure 2:**
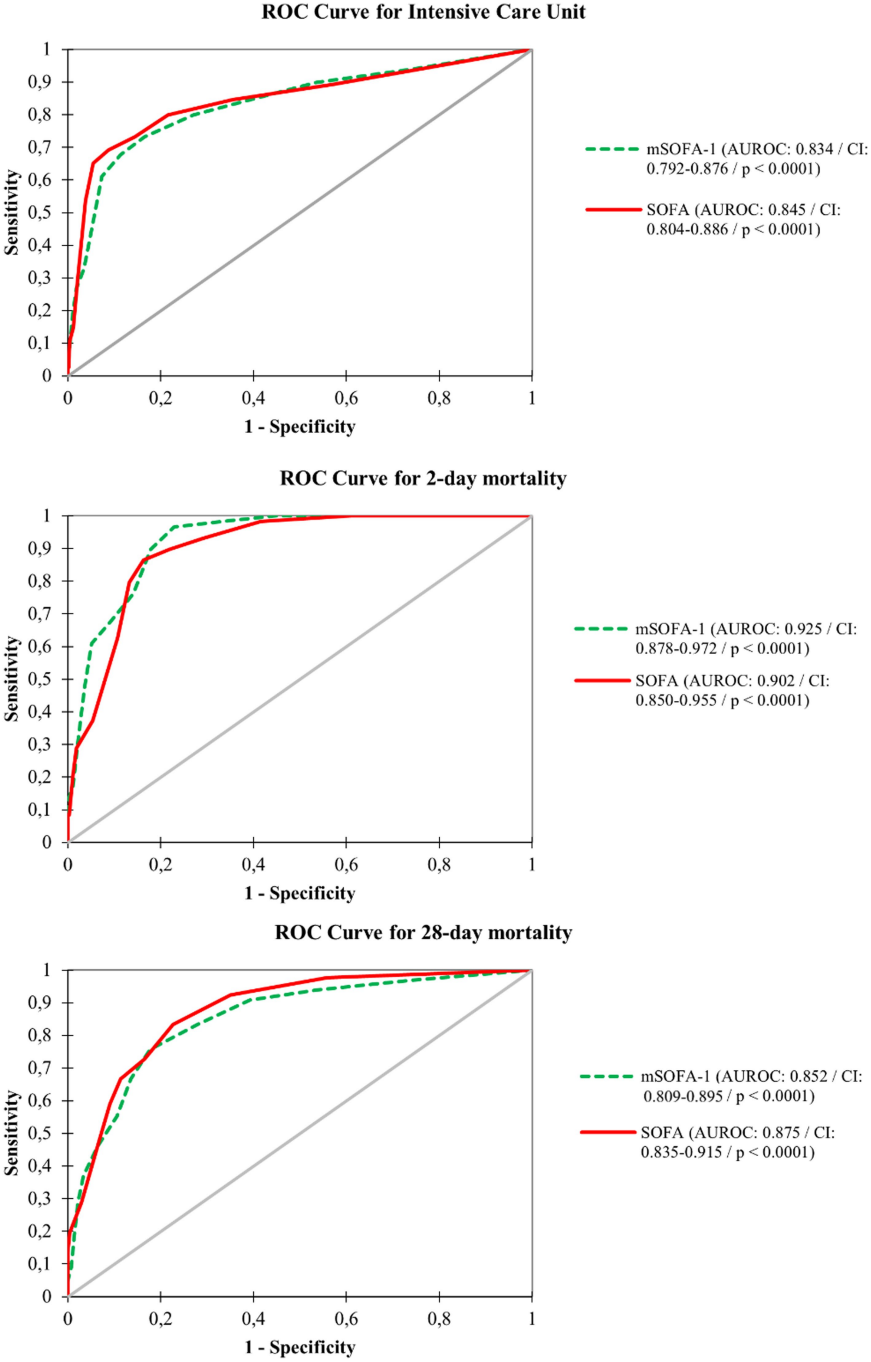
Diagnostic performance curves and areas under the curve for intensive care unit, two-day mortality and 28-day mortality. SOFA, Sequential Organ Failure Assessment; mSOFA, modified Sequential Organ Failure Assessment.

**Figure 3:**
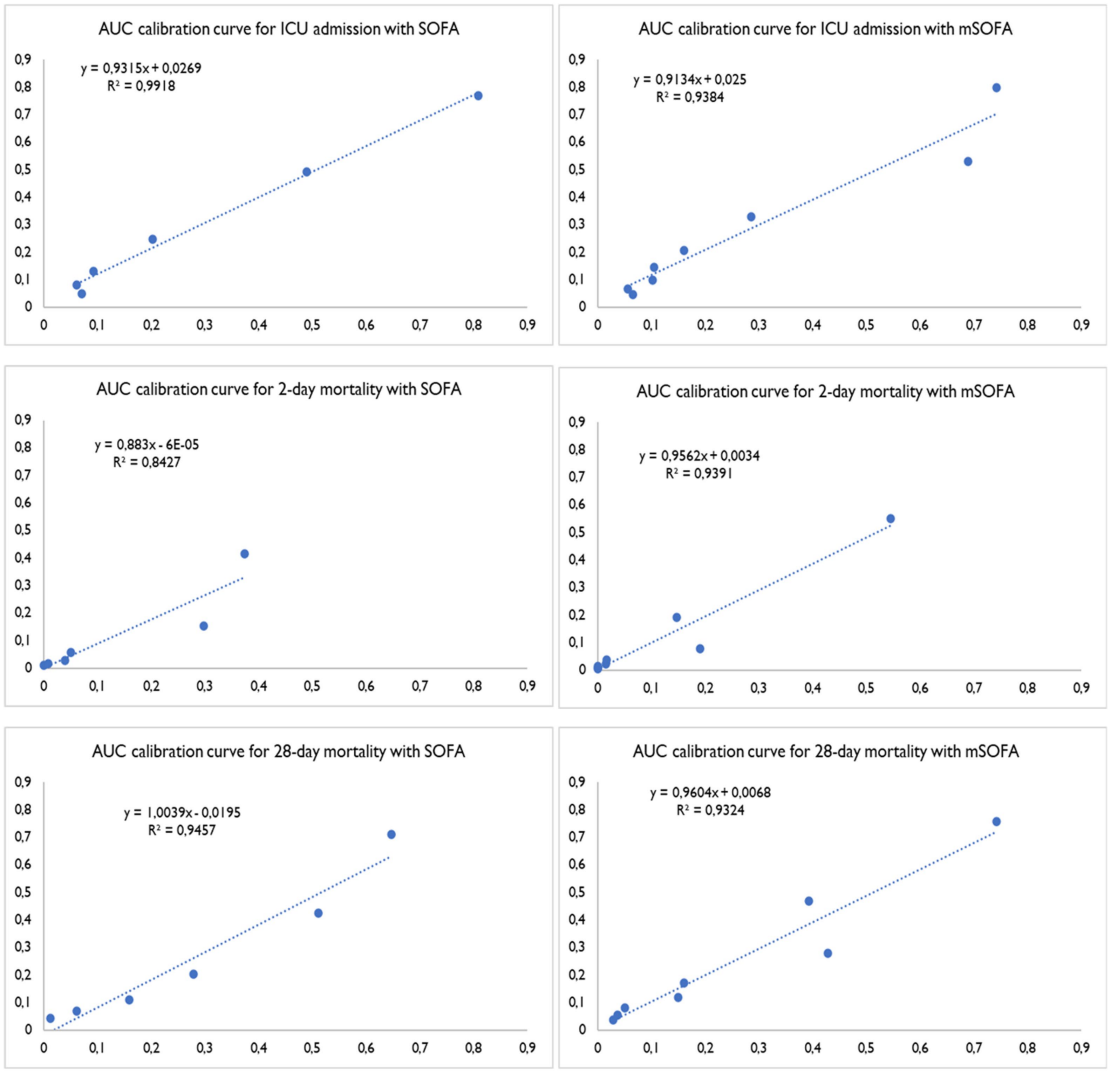
AUC calibration curves for intensive care unit care, two-day mortality and 28-day mortality. SOFA, Sequential Organ Failure Assessment; mSOFA, modified Sequential Organ Failure Assessment.

Figure 2 shows the discriminative power of the score for 2-day mortality, revealing a higher AUC for mSOFA (0.925 [95% CI: 0.878–0.972]) than for SOFA (0.902 [0.850–0.955]). Conversely, for 28-day mortality and the need for ICU admission, the SOFA score presented the largest AUC values at 28-day mortality: AUC of 0.875 [95% CI: 0.835–0.915]; and for ICU admission: AUC of 0.845 [95% CI: 0.804–0.886]. The results were supported by those resulting from the calibration curves (Figure 3), showing a better fit of mSOFA for 2-day mortality and a better fit of SOFA for ICU admission and 28-day mortality. Further details of the discriminative power can be found in Table 3.

**Table 3:** AUROC, cutoff points for combined sensitivity and specificity with best score (Youden’s test) for the different scores analyzed.

Scores	Nonsurvivors 2 days	Intensive care unit	Nonsurvivors 28 days
mSOFA			
Cutoff	5	5	5
AUROC	0.925 (0.878–0.972)	0.834 (0.792–0.876)	0.852 (0.809–0.895)
Sensitivity	96.6 (88.5–99.1)	73.2 (65.6–79.6)	75.8 (67.8–82.3)
Specificity	77.1 (73.5–80.3)	83.5 (80.0–86.5)	82.3 (78.7–85.4)
PPV	30.0 (23.9–36.9)	57.4 (50.3–64.2)	52.6 (45.6–59.6)
NPV	99.6 (98.4–99.9)	91.1 (88.1–93.4)	92.9 (90.1–94.9)
Likelihood ratio +	4.22 (3.61–4.94)	4.43 (3.55–5.53)	4.28 (3.46–5.28)
Likelihood ratio –	0.04 (0.01–0.17)	0.32 (0.24–0.42)	0.29 (0.22–0.40)
Odds ratio	96.00 (23.13–398.46)	13.79 (8.94–21.28)	14.51 (9.17–22.96)
Diagnostic accuracy	78.9 (75.6–81.9)	81.1 (77.9–83.9)	80.9 (77.7–83.8)
SOFA			
Cutoff	5	5	3
AUROC	0.902 (0.850–0.955)	0.845 (0.804–0.886)	0.875 (0.835–0.915)
Sensitivity	86.4 (75.5–93.0)	69.1 (61.3–76.0)	83.3 (76.1–88.7)
Specificity	83.6 (80.4–86.4)	91.2 (88.4–93.4)	77.4 (73.5–80.8)
PPV	34.9 (27.7–43.0)	70.5 (62.7–77.3)	48.9 (42.4–55.4)
NPV	98.4 (96.8–99.2)	90.7 (87.8–92.9)	94.7 (92.1–96.5)
Likelihood ratio +	5.29 (4.29–6.52)	7.89 (5.82–10.71)	3.68 (3.08–4.40)
Likelihood ratio –	0.16 (0.08–0.31)	0.34 (0.26–0.43)	0.22 (0.15–0.32)
Odds ratio	32.61 (14.99–70.94)	23.33 (14.61–37.24)	17.09 (10.34–28.25)
Diagnostic accuracy	83.9 (80.9–86.5)	86.1 (83.2–88.6)	78.6 (75.3–81.6)

Bracketed numbers indicate 95% confidence interval. SOFA, Sequential Organ Failure Assessment; mSOFA, modified Sequential Organ Failure Assessment; AUROC, area under the receiver operating characteristics; PPV, positive predictive value; NPV, negative predictive value.

## 4. Discussion

In this multicenter prospective cohort study, we analyzed the role of SOFA and mSOFA in the prediction of 2-day and 28-day mortality, as well as the requirement for ICU admission in a cohort of patients with acute neurological pathology, both showing excellent predictive value. However, some differences exist between them, with mSOFA being better for short-term mortality and SOFA for medium-term mortality and ICU admission prediction.

Both scores present a clear difference regarding their use. On the one hand, SOFA is a widespread and consolidated score that, although developed in 1996 to assess the prognosis of patients with sepsis-related multiorgan dysfunction (19), currently has 7 modifications according to the review conducted in 2023 by Xuesong Wang et al. (20) and numerous uses. On the other hand, mSOFA is a modern score scarcely implemented since it was developed in 2021. However, several studies have presented its clinical utility. mSOFA was used in an out-of-hospital setting in patients treated by emergency medical services, and the authors found an AUC of 0.946 for predicting 2-day mortality (all-cause) (14). Similar results for predicting 2-day mortality were found in the study by Castro Portillo et al. (21) (AUC = 0.943), which showed that mSOFA performed better than the other four scores: the TIMI risk index (TIMI), the modified shock index (MSI), the Cardiac Arrest Risk Triage (CART) and the National Early Warning Score 2 (NEWS2) for predicting 90-day mortality (in that study, the cohort was patients with acute cardiovascular disease). The study by Melero-Guijarro et al. (22) found that, in addition to 2-day mortality (AUC = 0.877), mSOFA was the best tool for predicting sepsis and septic shock (*vs* NEWS2 and qSOFA). Other works have compared SOFA with other mSOFA versions. One of them, in which mSOFA included hepatic and neurological SOFA criteria and information regarding chronic kidney disease and breathing support, showed that mSOFA performs similarly to SOFA (23). Another study in which mSOFA measured all the parameters from SOFA without the analytical parameters but including the pulse oximetry saturation/fraction of inspired oxygen ratio also performed similarly to SOFA (24).

As explained above, in the score calculation, SOFA and mSOFA have common items, including variables for neurological, cardiovascular, respiratory, and renal function assessment. Regarding the differences, SOFA is focused on coagulation and liver function through analysis of platelets and bilirubin in the laboratory. mSOFA adds lactate, a quick biomarker of anaerobic metabolism and a very specific predictor of poor short-term prognosis. This difference, which in fact could explain our results, supports the use of mSOFA in time-dependent pathologies with early deterioration and SOFA in clinical conditions with unexpected development.

This difference is important in the evaluation of neurological patients, since those patients commonly presented worse outcomes than patients with other conditions. Neurological conditions are generally time-dependent clinical situations in which rapid assessment, transfer, and intervention can be vital; these factors influence the characteristics of the score selected, not only in out-of-hospital care (25, 26) but also during the follow-up of their evolution (27, 28). The choice of an appropriate score is essential, as in some cases, they can be extremely sensitive to changes in the clinical condition (29). For instance, there is wide evidence in the literature of the use of EWS in conditions with a higher associated mortality, such as ischemic stroke or hemorrhage (15, 16, 30, 31). However, EWS also has no repercussions in pathologies whose recovery is associated with a restoration of clinical constants, such as seizures (32).

For these reasons, it is important that health professionals deepen their knowledge about tools to assess the clinical status of patients. This allows them to make evidence-based decisions according to the pathology identified. We consider that mSOFA, applied to neurological patients, who commonly present severe conditions, allows a global overview of patient status. In fact, the elevated discriminative power not only allows a proper tagging of patients at risk of deterioration but also, based on the recognition of patients with low risk, allows mSOFA to be used as a decision tool for patient admission. Moreover, the good identification of patient prognosis, evaluated here by the 28-day mortality outcome, allows an adequate selection of the follow-up protocol of patients.

## 5. Limitations

This study has several limitations that should be considered when interpreting the results. First, the sample selection was not random, and the data were not blinded, which can lead to bias in data selection. This point was minimized by having a multicentered sample and sufficiently clear inclusion criteria so that the opinion of the data extractor did not influence the final sample. In addition, the diagnosis of the clinical condition of acute neurological pathology was based on hospital anamnesis and on the clinical indicators recorded. Second, approximately 70% of the clinical cases studied corresponded to patients with seizures, ischemic stroke or hemorrhage, which limits the possible extrapolation of the results. Third, the study was carried out between 1 January 2019 and 31 August 2022, which means that the COVID-19 pandemic interfered with data collection, and the results obtained could have been affected. Fourth, dynamic evaluation of patient variables allows a better follow-up of patients; unfortunately, informatization of our EMS is not a reality, and therefore, we cannot benefit from the dynamic evaluation of patients. Fifth, although the study included a sufficient multicenter sample to obtain preliminary results, it would be beneficial to carry out additional studies on a wider score and in multiple centers to generalize the findings. Finally, the study reported a considerable percentage of seizures. This type of patient presents hyperacute values and parameters (e.g., lactate) in prehospital critical care, which should be interpreted with prudence. On the other hand, mortality in patients with seizures is usually low but not nonexistent; in fact, it tends to be higher when associated with other pathological conditions. In future studies, it may be pertinent to differentiate the outcomes in this particular cluster.

## 6. Conclusion

In summary, the results of this study present SOFA and mSOFA as adequate scores that should be considered for the prediction of 2-day and 28-day mortality (all-cause), as well as for ICU admission, in patients with acute neurological conditions. In particular, mSOFA should be considered when dealing with short-term outcomes, and SOFA should be considered for mid-term and ICU admission. The inclusion of these scores could improve early risk deterioration assessment and patient treatment.

## Data availability statement

The raw data supporting the conclusions of this article will be made available upon reasonable request by the corresponding author.

## Ethics statement

The studies involving humans were approved by Ref. CEIC 2049, MBCA/dgc, PI 18–895, Ref. CEIm PI010-18, PI 2018 10–119. The studies were conducted in accordance with the local legislation and institutional requirements. The participants provided their written informed consent to participate in this study.

## Author contributions

MD-C: Conceptualization, Data curation, Funding acquisition, Investigation, Validation, Visualization, Writing – original draft, Writing – review & editing. AS-G: Conceptualization, Data curation, Formal analysis, Funding acquisition, Methodology, Supervision, Validation, Visualization, Writing – original draft, Writing – review & editing. BP-L: Data curation, Formal analysis, Funding acquisition, Investigation, Methodology, Project administration, Resources, Software, Supervision, Writing – review & editing. CMM: Data curation, Formal analysis, Investigation, Methodology, Project administration, Resources, Software, Writing – review & editing. CDF: Formal analysis, Investigation, Methodology, Project administration, Resources, Software, Supervision, Writing – original draft, Writing – review & editing. LM-M: Formal analysis, Investigation, Methodology, Project administration, Resources, Writing – review & editing. AM-M: Data curation, Formal analysis, Investigation, Methodology, Project administration, Resources, Software, Supervision, Writing – review & editing. RC-S: Conceptualization, Data curation, Project administration, Resources, Validation, Visualization, Writing – review & editing. MO-A: Data curation, Formal analysis, Investigation, Methodology, Project administration, Resources, Software, Supervision, Validation, Visualization, Writing – review & editing. CJ-S: Formal analysis, Investigation, Project administration, Resources, Software, Supervision, Visualization, Writing – review & editing. JM-C: Data curation, Formal analysis, Investigation, Methodology, Project administration, Resources, Software, Supervision, Validation, Visualization, Writing – review & editing. FM-R: Validation, Writing – original draft, Writing – review & editing, Formal analysis, Funding acquisition, Investigation, Methodology, Project administration, Resources, Software, Supervision.

## Footnotes

^1^ https://doi.org/10.1186/ISRCTN48326533
