# Supplementary material for: Clinical outcome prediction of acute neurological patients admitted to the emergency department: Sequential Organ Failure Assessment score and modified SOFA score
Source: Front Public Health. 2023 Oct 30;11:1264159. doi: 10.3389/fpubh.2023.1264159 (PMC10642972; doi:10.3389/fpubh.2023.1264159)
Supplement: Supplementary file 1 [file Table_1.docx]

| Suppl. table 1: Comparison of patient variables recorded in the emergency department according to the patients’ intensive care. | | | | | | | | | | |
| --- | --- | --- | --- | --- | --- | --- | --- | --- | --- | --- |
| Variables ^1^ | | | | | Intensive-Care Unit | | | | p value and effect size ^2^ | |
|  |  |  |  |  | Yes | | No | |  |  |
| Number | | | | | 149 (23%) | | 491 (77%) | |  | |
| Demographic | | | | |  | |  | |  | |
|  | Age (years) | | | | 63 (52-76) | | 70 (52-83) | | p = 0.003* (0.12) ^T^ | |
|  | Sex | | | |  | |  | |  | |
|  | | | Male | | 91 (61%) | | 268 (55%) | | p = 0.16 | |
|  | | | Female | | 58 (39%) | | 223 (45%) | |  |  |
| Initial evaluation | | | | |  | |  | |  | |
|  | Pulse (bpm) | | | | 85 (70-100) | | 81 (69-95) | | p = 0.17 | |
|  | Respiratory rate (bpm) | | | | 15 (15-16) | | 16 (13-18) | | p = 0.45 | |
|  | Temperature (°C) | | | | 36.0 (35.6-36.6) | | 36.0 (35.8-36.5) | | p = 0.25 | |
|  | Systolic Blood Pressure (mmHg) | | | | 136 (112-165) | | 133 (117-151) | | p = 0.50 | |
|  | Diastolic Blood Pressure (mmHg) | | | | 77 (61-91) | | 76 (65-85) | | p = 0.57 | |
|  | Mean Blood Pressure (mmHg) | | | | 97 (79-118) | | 95 (84-106) | | p = 0.50 | |
|  | SpO_2_ (%) | | | | 98 (95-100) | | 97 (94-98) | | p < 0.001* (0.15) ^T^ | |
|  | SaFi | | | | 188 (99-415) | | 457 (433-467) | | p < 0.001* (0.45) ^S^ | |
|  | Fi O_2_ (%) | | | | 0.50 (0.24-0.99) | | 0.21 (0.21-0.21) | | p < 0.001* (0.56) ^M^ | |
|  | Glasgow Coma Scale (total) | | | | 3 (3-11) | | 15 (14-15) | | p < 0.001* (0.57) ^M^ | |
|  | | | Eye Opening Response | | 1 (1-3) | | 4 (3-4) | | p < 0.001* (0.57) ^M^ | |
|  | | | Verbal Response | | 1 (1-3) | | 5 (5-5) | | p < 0.001* (0.58) ^M^ | |
|  | | | Motor Response | | 1 (1-5) | | 6 (6-6) | | p < 0.001* (0.62) ^M^ | |
|  | | Lactate | | | 2.7 (1.6-4.6) | | 2.1 (1.5-3.9) | | p = 0.008* (0.11) ^T^ | |
|  | | Hematocrit | | | 41.6 (36.6-45.2) | | 41.0 (37.0-44.3) | | p = 0.55 | |
|  | | Leukocytes | | | 11.6 (8.3-15.4) | | 8.7 (6.7-11.2) | | p < 0.001* (0.25) ^S^ | |
|  | | Platelets | | | 210 (163-264) | | 215 (170-260) | | p = 0.51 | |
|  | | Glucose | | | 141 (119-189) | | 130 (108-168) | | p = 0.006* (0.11) ^T^ | |
|  | | Creatinine | | | 0.96 (0.74-1.26) | | 0.89 (0.74-1.12) | | p = 0.14 | |
|  | | Bilirubin | | | 0.51 (0.45-0.89) | | 0.51 (0.47-0.61) | | p = 0.08 | |
| Hospital Triage | | | | |  | |  | |  | |
|  | Level I: Resuscitation | | | | 84 (56%) | | 15 (3%) | | p < 0.001* (0.62) ^L^ | |
|  | Level II: Emergency | | | | 55 (37%) | | 221 (45%) | | p = 0.08 | |
|  | Level III: Urgency | | | | 10 (7%) | | 255 (52%) | | p < 0.001* (0.39) ^M^ | |
| Pathology | | | | |  | |  | |  | |
|  | | Seizures | | | 24 (16%) | | 162 (33%) | | p < 0.001* (0.16) ^S^ | |
|  | | Ischemic stroke | | | 16 (11%) | | 111 (23%) | | p = 0.001* (0.13) ^S^ | |
|  | | Hemorrhage | | | 71 (48%) | | 47 (10%) | | p < 0.001* (0.42) ^M^ | |
|  | | Infection | | | 13 (8%) | | 40 (8%) | | p = 0.82 | |
|  | | Confusion syndrome | | | 4 (3%) | | 40 (8%) | | p = 0.021* (0.09) ^T^ | |
|  | | Degenerative disease | | | 4 (3%) | | 19 (4%) | | p = 0.50 | |
|  | | Headache | | | 0 (0%) | | 21 (4%) | | p = 0.010* (0.10) ^S^ | |
|  | | Coma | | | 14 (9%) | | 7 (1%) | | p < 0.001* (0.19) ^S^ | |
|  | | Vertigo | | | 0 (0%) | | 18 (4%) | | p = 0.018* (0.09) ^T^ | |
|  | | Tumor | | | 3 (2%) | | 14 (3%) | | p = 0.58 | |
|  | | Neuromediated syncope | | | 0 (0%) | | 12 (2%) | | p = 0.06 | |
| Hospital interventions | | | |  | |  | |  | |  |
|  | | CT-scan | | | 148 (99%) | | 379 (77%) | | p < 0.001* (0.25) ^S^ | |
|  | | Ultrasound scan | | | 56 (38%) | | 94 (19%) | | p < 0.001* (0.18) ^S^ | |
|  | | Surgery | | | 38 (26%) | | 3 (1%) | | p < 0.001* (0.43) ^M^ | |
|  | | Coronary/neurovascular interv. | | | 29 (20%) | | 28 (6%) | | p < 0.001* (0.20) ^S^ | |
| Hospital outcomes | | | | |  | |  | |  | |
|  | Inpatients | | | | 148 (99%) | | 310 (63%) | | p < 0.001* (0.34) ^M^ | |
|  | Hospitalization days (inpatients) | | | | 10 (3-20) | | 6 (3-11) | | p = 0.005* (0.13) ^T^ | |
|  | ICU days (ICU inpatients) | | | | 4 (2-9) | | - | | - | |
| Mortality | | | | |  | |  | |  | |
|  | Day 2 | | | | 36 (24%) | | 23 (5%) | | p < 0.001* (0.29) ^S^ | |
|  | Day 28 | | | | 71 (48%) | | 61 (12%) | | p < 0.001* (0.37) ^M^ | |
| EWS analyzed | | | | |  | |  | |  | |
|  | mSOFA | | | | 7 (4-10) | | 2 (0-4) | | p < 0.001* (0.49) ^S^ | |
|  | SOFA | | | | 7 (3-8) | | 1 (0-2) | | p < 0.001* (0.52) ^M^ | |
| ^1^ Values expressed as a total number (fraction) and medians (1^st^ quartile-3^rd^ quartile) as appropriate. Bracketed numbers indicate 95% confidence interval. ^2^ The p values were calculated with the Mann‒Whitney U test and Chi square test. Effect Size were calculated with the Rosenthal r test [Trivial ^(T)^ (< 0.2); Small ^(S)^ (0.2 - 0.5); Moderate ^(M)^ (0.5 - 0.8); Large ^(L)^ (0.8 - 1.3); Very Large ^(VL)^ (≥ 1.3)] and Cramer V test [Trivial ^(T)^ (< 0.1); Small ^(S)^ (0.1 - 0.3); Medium ^(M)^ (0.3 - 0.5); Large ^(L)^ ≥ 0.5]. SpO_2_: Oxygen saturation; SaFi: pulse oximetry saturation/fraction of inspired oxygen ratio; CT-scan: computerized axial tomography. SOFA: Sequential Organ Failure Assessment; mSOFA: modified Sequential Organ Failure Assessment. | | | | | | | | | | |

| Suppl. table 2: Comparison of patient variables recorded in the emergency department according to 28-day mortality. | | | | | | |
| --- | --- | --- | --- | --- | --- | --- |
| Variables ^1^ | | | | Survivors | Nonsurvivors 28 days | p value and effect size ^2^ |
| Number | | | | 508 (79%) | 132 (21%) | - |
| Demographic | | | |  |  |  |
|  | Age (years) | | | 64 (50-78) | 79 (64-86) | p < 0.001* (0.26) ^S^ |
|  | Sex | | |  |  |  |
|  | | | Male | 283 (56%) | 76 (58%) | p = 0.70 |
|  | | | Female | 225 (44%) | 56 (42%) |  |
| Initial evaluation | | | |  |  |  |
|  | Pulse (bpm) | | | 83 (70-95) | 82 (69-97) | p = 0.73 |
|  | Respiratory rate (bpm) | | | 15 (13-18) | 15 (15-19) | p = 0.005* (0.11) ^T^ |
|  | Temperature (°C) | | | 36.1 (35.8-36.5) | 36.0 (35.4-36.6) | p = 0.004* (0.11) ^T^ |
|  | Systolic Blood Pressure (mmHg) | | | 133 (116-150) | 144 (120-167) | p = 0.006* (0.11) ^T^ |
|  | Diastolic Blood Pressure (mmHg) | | | 76 (65-85) | 78 (60-92) | p = 0.28 |
|  | Mean Blood Pressure (mmHg) | | | 94 (84-105) | 100 (83-119) | p = 0.041* (0.08) ^T^ |
|  | SpO_2_ (%) | | | 97 (95-99) | 96 (93-99) | p = 0.08 |
|  | SaFi | | | 457 (425-467) | 194 (99-438) | p < 0.001* (0.41) ^S^ |
|  | Fi O_2_ (%) | | | 0.21 (0.21-0.21) | 0.50 (0.21-0.99) | p < 0.001* (0.46) ^S^ |
|  | Glasgow Coma Scale (total) | | | 15 (14-15) | 4 (3-11) | p < 0.001* (0.55) ^M^ |
|  | | | Eye Opening Response | 4 (3-4) | 1 (1-3) | p < 0.001* (0.51) ^M^ |
|  | | | Verbal Response | 5 (5-5) | 1 (1-3) | p < 0.001* (0.55) ^M^ |
|  | | | Motor Response | 6 (6-6) | 2 (1-5) | p < 0.001* (0.56) ^M^ |
|  | | Lactate | | 2.1 (1.4-3.7) | 2.9 (1.7-5.6) | p < 0.001* (0.15) ^T^ |
|  | | Hematocrit | | 41.4 (37.5-45.0) | 39.5 (35.0-43.6) | p = 0.003* (0.12) ^T^ |
|  | | Leukocytes | | 8.9 (6.9-11.4) | 11.0 (7.8-14.9) | p < 0.001* (0.17) ^T^ |
|  | | Platelets | | 215 (170-260) | 211 (164-268) | p = 0.56 |
|  | | Glucose | | 128 (107-164) | 150 (125-192) | p < 0.001* (0.19) ^T^ |
|  | | Creatinine | | 0.87 (0.73-1.09) | 1.10 (0.77-1.63) | p < 0.001* (0.19) ^T^ |
|  | | Bilirubin | | 0.51 (0.45-0.62) | 0.51 (0.48-0.89) | p = 0.017* (0.09) ^T^ |
| Hospital Triage | | | |  |  |  |
|  | Level I: Resuscitation | | | 46 (9%) | 53 (40%) | p < 0.001* (0.35) ^M^ |
|  | Level II: Emergency | | | 212 (42%) | 64 (49%) | p = 0.16 |
|  | Level III: Urgency | | | 250 (49%) | 15 (11%) | p < 0.001* (0.31) ^M^ |
| Pathology | | | |  |  |  |
|  | | Seizures | | 180 (35%) | 6 (4%) | p < 0.001* (0.28) ^S^ |
|  | | Ischemic stroke | | 100 (20%) | 27 (21%) | p = 0.84 |
|  | | Hemorrhage | | 56 (11%) | 62 (47%) | p < 0.001* (0.38) ^M^ |
|  | | Confusion syndrome | | 40 (8%) | 4 (3%) | p = 0.050* (0.08) ^T^ |
|  | | Infection | | 35 (7%) | 18 (14%) | p = 0.012* (0.10) ^S^ |
|  | | Headache | | 21 (4%) | 0 (0%) | p = 0.018* (0.09) ^T^ |
|  | | Degenerative disease | | 21 (4%) | 2 (1%) | p = 0.15 |
|  | | Vertigo | | 18 (4%) | 0 (0%) | p = 0.028* (0.09) ^T^ |
|  | | Tumor | | 14 (3%) | 3 (2%) | p = 0.76 |
|  | | Neuromediated syncope | | 12 (2%) | 0 (0%) | p = 0.08 |
|  | | Coma | | 11 (2%) | 10 (8%) | p = 0.002* (0.12) ^M^ |
| Hospital interventions | | | |  | | |
|  | | CT-scan | | 407 (80%) | 120 (91%) | p = 0.004* (0.12) ^S^ |
|  | | Ultrasound scan | | 117 (23%) | 33 (25%) | p = 0.63 |
|  | | Surgery | | 27 (5%) | 14 (11%) | p = 0.027* (0.09) ^T^ |
|  | | Coronary/neurovascular interv. | | 43 (9%) | 14 (11%) | p = 0.44 |
| Hospital outcomes | | | |  |  |  |
|  | Inpatients | | | 328 (65%) | 130 (99%) | p < 0.001* (0.30) ^M^ |
|  | Hospitalization days (inpatients) | | | 8 (5-14) | 3 (1-9) | p < 0.001* (0.31) ^S^ |
|  | Intensive care unit | | | 78 (15%) | 71 (54%) | p < 0.001* (0.37) ^M^ |
|  | ICU days (ICU inpatients) | | | 5 (3-10) | 2 (1-8) | p = 0.001* (0.27) ^S^ |
| EWS analyzed | | | |  |  |  |
|  | mSOFA | | | 2 (0-4) | 7 (5-10) | p < 0.001* (0.50) ^M^ |
|  | SOFA | | | 1 (0-2) | 6 (3-8) | p < 0.001* (0.54) ^M^ |
| ^1^ Values expressed as a total number (fraction) and medians (1^st^ quartile-3^rd^ quartile) as appropriate. Bracketed numbers indicate 95% confidence interval. ^2^ The p values were calculated with the Mann‒Whitney U test and Chi square test. Effect Size were calculated with the Rosenthal r test [Trivial ^(T)^ (< 0.2); Small ^(S)^ (0.2 - 0.5); Moderate ^(M)^ (0.5 - 0.8); Large ^(L)^ (0.8 - 1.3); Very Large ^(VL)^ (≥ 1.3)] and Cramer V test [Trivial ^(T)^ (< 0.1); Small ^(S)^ (0.1 - 0.3); Medium ^(M)^ (0.3 - 0.5); Large ^(L)^ ≥ 0.5]. SpO_2_: Oxygen saturation; SaFi: pulse oximetry saturation/fraction of inspired oxygen ratio; CT-scan: computerized axial tomography. SOFA: Sequential Organ Failure Assessment; mSOFA: modified Sequential Organ Failure Assessment. | | | | | | |
